# Supplementary material for: Wavefront dislocations reveal the topology of quasi-1D photonic insulators
Source: Nat Commun. 2021 Jun 11;12:3571. doi: 10.1038/s41467-021-23790-w (PMC8196168; doi:10.1038/s41467-021-23790-w)
Supplement: Supplementary file 1 — Supplementary Information [file 41467_2021_23790_MOESM1_ESM.pdf]

Supplementary Information: Wavefront dislocations reveal the topology of quasi-1D photonic insulators

Clément Dutreix,[1,*] Matthieu Bellec,[2] Pierre Delplace,[3] and Fabrice Mortessagne[2,†]

[1]*Université de Bordeaux, France and CNRS, LOMA, UMR 5798, F-33400 Talence, France*
[2]*Université Côte d'Azur, CNRS, Institut de Physique de Nice, 06100 Nice, France*
[3]*Univ Lyon, Ens de Lyon, Univ Claude Bernard, CNRS, Laboratoire de Physique, F-69342 Lyon, France*

Supplementary Note 1. MICROWAVE REALISATION OF TIGHT-BINDING SYSTEMS

The experimental setup is designed to realise a microwave system that is well approximated by a nearest-neighbour tight-binding description [1]. The sites of the lattice are occupied by dielectric microwave resonators with a cylindrical shape and made of ZrSnTiO ceramics (Temex-Ceramics, E8000 series: 5 mm height, 3 mm radius and a refractive index $n_r \approx 6$) sandwiched between two metallic plates at a distance $h = 16$ mm. Each resonator supports a fundamental TE mode of bare frequency of 7.435 GHz, which corresponds to the on-site energy of atoms in a tight-binding model. As the resonance frequency is below the cut-off frequency of the first TE mode defined by the two plates the adjacent resonators are coupled through evanescent wave components leading to an approximately exponential decay of the coupling strength t with the distance between the resonators [1]. The system is excited via a loop antenna fixed in the movable top plate thus allowing to scan spatially the magnetic field B_z, which is the only magnetic field component for this mode. From the reflection measurements performed by a vector network analyzer (ZVA 24 from Rohde & Schwarz) the local density of states can be extracted (for details see Ref. [1]) and finally, by integrating over space, the density of states. In all these experiments, we face an intrinsic on-site disorder of $\sim 0.15\%$ in the values of the bare frequency.

Supplementary Note 2. EXPERIMENTAL LDOS MAPS

In the manuscript, we focus specifically on the LDOS fluctuations induced by the leftmost edge of the SSH chain (see Manuscript Fig. 1b). Here, we present in Supplementary Fig. 1 the LDOS of the whole SSH chain. It shows that the discussions in the manuscript also hold for the rightmost edge, thus exemplifying space inversion symmetry. For instance, we observe that the shift in the number of interference fringes on sublattice A close to the leftmost edge also occurs on sublattice B close to the rightmost edge (compare e.g. the inversion symmetric fringes in $\rho_A(m = 6)$ and $\rho_B(m = 17)$ in Supplementary Fig. 1).

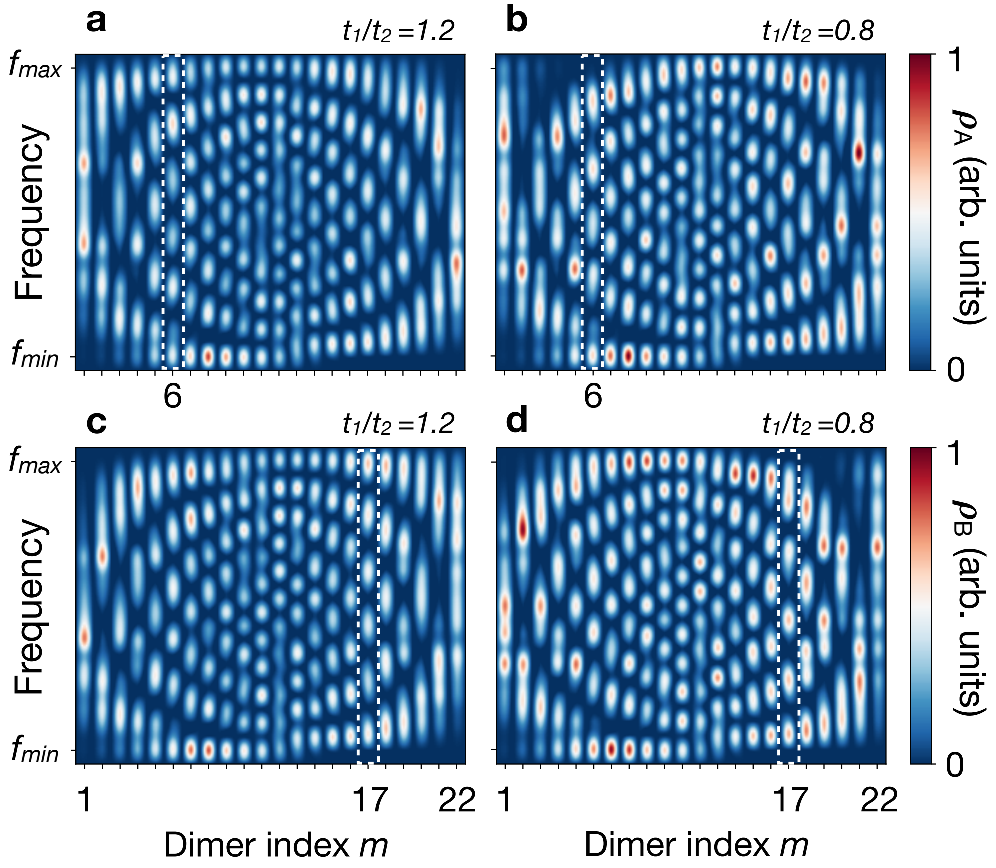

Supplementary Fig. 1. **LDOS measured in the SSH chain made of 44 dielectric resonators**: **a**, LDOS of the lower band measured on sublattice A in the trivial regime ($t_1/t_2 = 1.2$). **b**, LDOS of the lower band measured on sublattice A in the topological regime ($t_1/t_2 = 0.8$). **c**, LDOS of the lower band measured on sublattice B in the trivial regime ($t_1/t_2 = 1.2$). **d**, LDOS of the lower band measured on sublattice B in the topological regime ($t_1/t_2 = 0.8$).

Supplementary Note 3. THEORETICAL DESCRIPTION OF THE SSH CHAIN

3.1. Bulk-boundary correspondence at the leftmost edge

3.1.1. Bulk topology

The winding number w introduced in the manuscript characterises the bulk topology of the insulator in the 1D Brillouin zone. It can be written equivalently as

$$w = \frac{1}{2i\pi} \oint_C dz \frac{h'(z)}{h(z)} , \tag{1}$$

where $z = e^{ik}$ and C is the unit circle centred on the origin of the complex plane. We can evaluated this integral through Cauchy's argument principle

$$w = r - p , \tag{2}$$

where r and p are respectively the numbers of zeros and poles of the function $h(z)$ inside the unit circle. Since $h(z) = (t_1 z + t_2)z^{-1}$ (cf. Eq. (1) in the manuscript), $z = 0$ is the single pole inside C and so $p = 1$. The number of zeros inside C depends on the values of the coupling strengths t_1 and t_2. Given the expression of $h(z)$, we find either $r = 1$ when $t_1 > t_2$, or $r = 0$ when $t_1 < t_2$. Thus, the winding number can take two different values

$$\begin{aligned} w_> &= r - 1 = 1 - 1 = 0 \quad \text{when} \quad t_1 > t_2 \\ w_< &= r - 1 = 0 - 1 = -1 \quad \text{when} \quad t_1 < t_2 . \end{aligned} \tag{3}$$

This characterises two distinct topological regimes for the wave functions delocalised through the bulk of the insulator, as introduced in the manuscript.

3.1.2. Midgap boundary modes

The chiral symmetry enforces the midgap modes to be sublattice polarised at zero energy. Assuming nearest-neighbour coupling, the wave functions of such modes satisfy independent recursive equations

$$t_1 A_m + t_2 A_{m+1} = 0 \quad \text{and} \quad t_1 B_m + t_2 B_{m-1} = 0 , \tag{4}$$

where A_m (resp. B_m) is the wave-function weight in unit cell m on sublattice A (resp. B). These recursive relations respectively have the characteristic equations

$$h(1/z) = 0 \quad \text{and} \quad h(z) = 0 . \tag{5}$$

Thus, the number of evanescent wave-functions on sublattice A (resp. B) is given by the number of zeros of $h(1/z)$ (resp. $h(z)$) inside the unit circle C. The polynomial h is of first order and has a single zero. So we find that there is $1 - r$ (resp. r) midgap boundary mode on sublattice A (resp. B). Nevertheless, the open boundary condition at the leftmost edge is $B_0 = 0$ (see Supplementary Fig. 2), which further prohibits the boundary mode on sublattice B. This demonstrates the bulk-boundary correspondence discussed in the manuscript at the leftmost edge: there is $\mathcal{N}_A = 1 - r \equiv -w$ (from Supplementary Eq. (3)) boundary mode polarised on sublattice A.

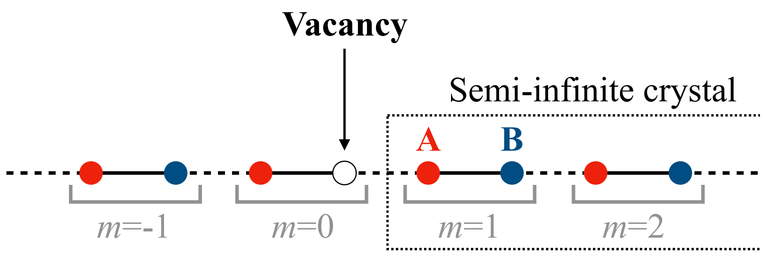

Supplementary Fig. 2. **Modelisation of the edge**: Within a nearest-neighbour tight-binding description, the edge is regarded as a vacancy breaking the translation invariance of the infinite crystal. We then focus on the semi-infinite crystal associated with positive dimer indices ($m > 0$).

3.2. Backscattering of the delocalised waves on the edge

The Bloch Hamiltonian of the SSH model is bipartite and reads

$$H(k) = \begin{pmatrix} 0 & h(k) \\ h^{\dagger}(k) & 0 \end{pmatrix} \tag{6}$$

in the sublattice basis (A, B). The single-particle Green function of the system with translation invariance satisfies

$$G(m,n) = \int_{-\pi}^{\pi} \frac{dk}{2\pi} \left[\omega \, \mathrm{I}_2 - H(k)\right]^{-1} e^{ik(m-n)} , \tag{7}$$

where the integers m and n label the dimers, and I_2 is the 2×2 identity matrix. We describe the edge as a vacancy and model it by a large on-site potential $V_B \, \delta_{m,0}$ on sublattice B (see Supplementary Fig. 2). We focus on the standing-wave interference it induces on dimers $m > 0$. Summing all the scattering orders in the potential V_B leads to the T-matrix

$$T = \begin{pmatrix} 0 & 0 \\ 0 & T_{BB} \end{pmatrix} \delta_{m,0} , \tag{8}$$

where $T_{BB} = -1/G_{BB}(m,m)$ in the limit $V_B \to \infty$. The sublattice components of the Green function that describes the backscattering of the delocalised Bloch waves on the edge satisfy

$$\begin{aligned} \mathcal{G}_{AA}(m,m) &= G_{AA}(m,m) + G_{AB}(m,0)\, T_{BB}(0,0)\, G_{BA}(0,m) = -iC(k)\left(1 + e^{i2km + i\delta_A(k) + i\pi}\right) \\ \mathcal{G}_{BB}(m,m) &= G_{BB}(m,m) + G_{BB}(m,0)\, T_{BB}(0,0)\, G_{BB}(0,m) = -iC(k)\left(1 + e^{i2km + i\pi}\right) , \end{aligned} \tag{9}$$

where $0 < k < \pi$ and $C(k) = -\omega t_2/(2t_1 \sin(k))$ is a positive coefficient for the lower band ($\omega < 0$). Backscattering on the leftmost edge involves the additional phase shift $\delta_A(k) = 2\mathrm{Arg}[h(k)]$ on sublattice A. The LDOS verifies $\rho = -\mathrm{Im}\,\mathcal{G}/\pi$, so its space

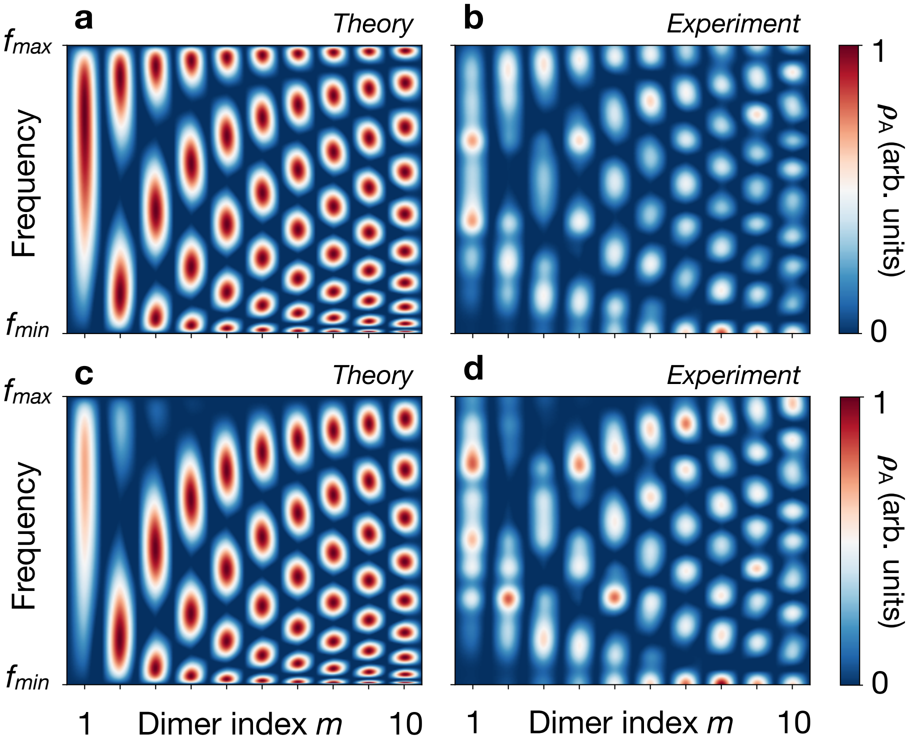

Supplementary Fig. 3. **Theoretical and experimental LDOS maps**: **a**, Space behaviour of the LDOS on sublattice A predicted from Supplementary Eq. (10) for the lower band in the trivial regime ($t_1/t_2 = 1.2$). **b**, LDOS of the lower band measured on sublattice A in the trivial regime ($t_1/t_2 = 1.2$). **c**, Space behaviour of the LDOS on sublattice A predicted from Supplementary Eq. (10) for the lower band in the topological regime ($t_1/t_2 = 0.8$). **d**, LDOS of the lower band measured on sublattice A in the topological regime ($t_1/t_2 = 0.8$).

behaviour consists of

$$\rho_A(m,\mathbf{s}) \propto 1 + \cos\left(2km + \delta_A(\mathbf{s}) + \pi\right) \qquad (10)$$
$$\rho_B(m,\mathbf{s}) \propto 1 + \cos\left(2km + \pi\right)\ ,$$

where we have introduced the vector $\mathbf{s} = (t_1/t_2, k)$. These sublattice-polarised expressions of the LDOS are the ones introduced in the manuscript. Supplementary Figure 3 shows that the space behaviour of the LDOS in Supplementary Eq. (10) describes well the one observed in the 1D photonic insulator in our experiment.

The wavefront dislocation discussed in the manuscript arises from the LDOS fluctuations $\Delta\rho_A = -\mathrm{Im}\,\Delta G_{AA}/\pi$, as a phase singularity of the scattering Green function

$$\Delta G_{AA}(m,m) = G_{AB}(m,0)\,T_{BB}(0,0)\,G_{BA}(0,m) \propto -ie^{i2km+i\delta_A(k)+i\pi}\ . \qquad (11)$$

This is the expression of the scattering Green function we introduce in the manuscript to explain the origin the LDOS dislocation.

Supplementary Note 4. GENERALISATION TO LARGER WINDING NUMBERS

Here, we extend beyond the SSH model the relevance of LDOS topological defects as new manifestation of the nontrivial bulk of 1D insulators. We first present how to experience 1D insulators with winding numbers larger than one through a nearest-neighbour tight-binding model. We derive a bulk-boundary correspondence showing that the winding number still provides the number of midgap edge states. We then describe the elastic backscattering of the delocalised waves on the edge of the system and reach similar conclusions as for the SSH insulator. The number of interference fringes in the LDOS still exhibits a wavefront dislocation whose strength reveals the larger winding number of the band structure. We finally realise such a system in our microwave experiment, which demonstrates that our approach is further relevant to measure topological indices larger than one.

4.1. Bulk-boundary correspondence at the leftmost edge

4.1.1. Bulk topology

One possibility to experience larger winding numbers in a 1D insulator is to consider additional coupling processes between distant neighbours in the SSH chain [2,3]. However, this is not possible in our microwave platform. The dielectric resonators couple each other through their evanescent fundamental TE modes, which mainly couples nearest-neighbour resonators. Now we show that this is nonetheless possible to experience larger winding numbers within a nearest-neighbour tight-binding description, as represented in Fig. 4 and Fig. 5 in the main manuscript. In the sublattice basis (A_1, A_2, B_1, B_2), the bipartite Bloch Hamiltonian reads

$$H(k) = \begin{pmatrix} 0 & h(k) \\ h^\dagger(k) & 0 \end{pmatrix}, \quad \text{where } h(k) = \begin{pmatrix} h_1(k) & 0 \\ t_\perp & h_2(k) \end{pmatrix} \qquad (12)$$

and $h_1(k) = t_1 + t_2 e^{-ik}$, $h_2(k) = t_3 + t_4 e^{-ik}$, while the dimensionless momentum verifies $-\pi \leq k \leq \pi$ and $t_i > 0$. There are four frequency bands associated with the dispersion relations $\pm f_1(k)$ and $\pm f_2(k)$. They satisfy

$$2\,f_1(k)^2 = |h_1(k)|^2 + |h_2(k)|^2 + t_\perp^2 - \sqrt{\Delta(k)}$$
$$2\,f_2(k)^2 = |h_1(k)|^2 + |h_2(k)|^2 + t_\perp^2 + \sqrt{\Delta(k)}\ , \qquad (13)$$

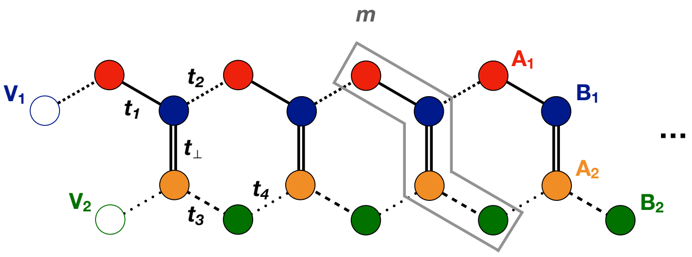

Supplementary Fig. 4. **Two coupled SSH chains**: There are four sublattices A_1, B_1, A_2, and B_2 per unit cell m. They are connected through the coupling strengths t_1, t_2, t_3, t_4, and $t_\perp$.The onsite potentials V_1 and V_2 model the edge within the nearest-neighbour tight-binding description.

where $\Delta(k) = (|h_1(k)|^2 + |h_2(k)|^2 + t_\perp^2)^2 - 4|h_1(k)|^2|h_2(k)|^2$. The bulk gap between the frequency bands f_1 and f_2 cannot close because $\Delta(k) > 0$ for $t_\perp \neq 0$. Only can the gap close at zero frequency when $\pm f_1 = 0$. This occurs at $k = \pi$ for $t_1 = t_2$ or $t_3 = t_4$. When $t_1 \neq t_2$ and $t_3 \neq t_4$, the Bloch Hamiltonian $H(k)$ defines a mapping from the 1D Brillouin zone S^1 to the space of gapped Hamiltonians, which reduces to S^1 under chiral symmetry. The first homotopy group of spheres $\pi_1(S^1) = \mathbb{Z}$ then characterises the band structure topology. We find that the topological index is a winding number w satisfying

$$w = \frac{1}{2i\pi} \oint_{\mathrm{BZ}} dk \, \nabla_k \ln \mathrm{Det}\, h(k) = \frac{1}{2i\pi} \sum_{n=1}^{2} \oint_C dz \frac{h_n'(z)}{h_n(z)} = \sum_{n=1}^{2} r_n - p_n = \sum_{n=1}^{2} w_n \,, \tag{14}$$

where $z = e^{ik}$ and r_n (p_n) denotes the number of zeros (poles) of h_n inside the unit circle C. Therefore, the topological index of the two coupled SSH chains is nothing but the sum of the individual winding numbers of each uncoupled SSH chain. From our previous study of the SSH chain, we readily identify three distinct topological regimes:

$$\begin{aligned} w &= 0 \quad \text{when} \quad t_1 > t_2 \quad \text{and} \quad t_3 > t_4 \\ w &= -1 \quad \text{when} \quad t_1 < t_2 \quad \text{and} \quad t_3 > t_4 \quad \text{or} \quad t_1 > t_2 \quad \text{and} \quad t_3 < t_4 \\ w &= -2 \quad \text{when} \quad t_1 < t_2 \quad \text{and} \quad t_3 < t_4 \,. \end{aligned} \tag{15}$$

Note that this analysis can be generalised straightforwardly to the case of N coupled SSH chains following the pattern in Supplementary Fig. 4, which then allows arbitrary winding numbers up to $w = N$.

4.1.2. Midgap boundary modes

Under chiral symmetry, the midgap modes are sublattice polarised at zero frequency. Assuming nearest-neighbour coupling, the wave functions of such modes satisfy independent recursive equations

$$\begin{aligned} t_1 A_1(m) + t_2 A_1(m+1) + t_\perp A_2(m) &= 0 \\ t_3 A_2(m) + t_4 A_2(m+1) &= 0 \,, \end{aligned} \tag{16}$$

where $A_n(m)$ is the wave-function weight in unit cell m on sublattice A_n. The second equation leads to the number of evanescent wave-functions on sublattice A_2. Following our previous analysis of the SSH chain, it is given by the number of zeros of $h_2(1/z)$ inside the unit circle C. Thus, we find that there is $\mathcal{N}_{A_2} = 1 - r_2 = -w_2$ midgap boundary mode on sublattice A_2. From the knowledge of $A_2(m)$, we can then determine the number of evanescent solutions on sublattice A_1 satisfying Supplementary Eq. (16). The homogeneous solution $A_{1,\mathrm{h}}(m)$ is associated with the characteristic equation $h_1(1/z) = 0$. Following our previous analysis of the SSH chain, it leads to $1 - r_1 = -w_1$ evanescent modes. The peculiar solution is of the form $A_{1,\mathrm{p}}(m) \propto A_2(m)$ and so describes $-w_2$ extra evanescent modes on sublattice A_1. In the end, the full solution is $A_1(m) = A_{1,\mathrm{h}}(m) + A_{1,\mathrm{p}}(m)$. Therefore, the number of independent evanescent modes localised at the leftmost edge on sublattice A_1 is

$$\mathcal{N}_{A_1} = -w_1 - w_2 = -w \,. \tag{17}$$

On the other hand, the zero-frequency modes polarised on sublattice B_n satisfy the recurrence relations

$$\begin{aligned} t_1 B_1(m) + t_2 B_1(m-1) &= 0 \\ t_\perp B_1(m) + t_3 B_2(m) + t_4 B_2(m-1) &= 0 \,, \end{aligned} \tag{18}$$

where $B_n(m)$ is the wave-function weight in unit cell m on sublattice B_n. A similar analysis as for the sublattices $\mathrm{A}_{1,2}$ readily shows that there is no evanescent mode localised at the leftmost edge on sublattice B_n satisfying the boundary condition $B_n(0) = 0$. This proves the bulk-boundary correspondence: the number of zero-energy edge states polarised on sublattices A_1 is given by the topological invariant of the bulk band structure $\mathcal{N}_{A_1} = -w$.

4.2. Effective third-nearest-neighbour tight-binding model

We can show that the low-frequency physics of our nearest-neighbour tight-binding microwave insulator realises an effective 1D insulator coupling resonators up to the third-nearest neighbours on sublattices A_1 and B_2. Indeed, the Bloch Hamiltonian in Supplementary Eq. (12) leads to the set of secular equations

$$\begin{aligned} h_1(k)\, B_1 &= f\, A_1 \\ h_1^*(k)\, A_1 + t_\perp A_2 &= f\, B_1 \\ t_\perp\, B_1 + h_2(k)\, B_2 &= f\, A_2 \\ h_2^*(k)\, A_2 &= f\, B_2 \,. \end{aligned} \tag{19}$$

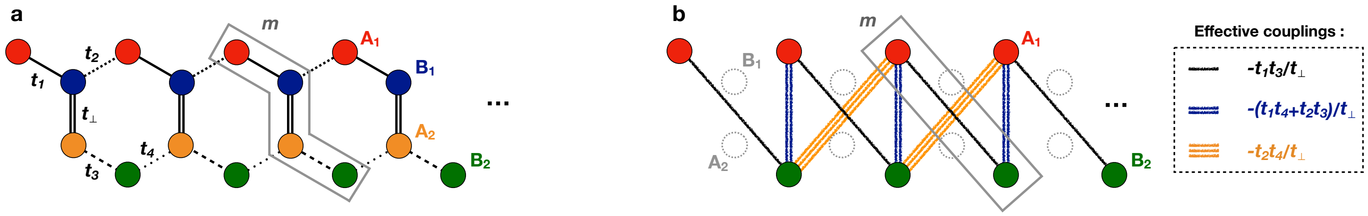

Supplementary Fig. 5. **Effective low-energy lattice model: a**, Microwave insulator described by the four-band Bloch Hamiltonian in Supplementary Eq. (12). **b**, Low-frequency physics of the same microwave insulator whose low-frequency physics described by the two-band Bloch Hamiltonian in Supplementary Eq. (21) with effective couplings involving up to the third nearest unit-cells.

We can rewrite the two middle equations as

$$t_\perp\left(1-\frac{f}{t_\perp}\right)A_2+t_\perp\left(1-\frac{f}{t_\perp}\right)B_1=-h_1^*(k)\,A_1-h_2(k)\,B_2$$
$$t_\perp\left(1+\frac{f}{t_\perp}\right)A_2-t_\perp\left(1+\frac{f}{t_\perp}\right)B_1=-h_1^*(k)\,A_1+h_2(k)\,B_2\,.$$

In the limit $f \ll t_\perp$, they reduce to

$$B_1 \simeq -\frac{h_2(k)}{t_\perp}B_2 \quad \text{and} \quad A_2 \simeq -\frac{h_1^*(k)}{t_\perp}A_1\,. \tag{20}$$

We can substitute them into Supplementary Eq. (19), so that the low-frequency band structure is described by the effective Bloch Hamiltonian

$$H_{\text{eff}}(k)=\begin{pmatrix}0 & -\frac{h_1(k)h_2(k)}{t_\perp}\\ -\frac{h_1^*(k)h_2^*(k)}{t_\perp} & 0\end{pmatrix}, \tag{21}$$

in the sublattice basis (A_1, B_2). Taking the Fourier transform readily shows that the low-frequency physics of the four-sublattice insulator in Supplementary Fig 5a only involves two sublattices within a third nearest-neighbour tight-binding description, as presented in Supplementary Fig 5b. We can also verify that the band-structure topology remains *exactly* captured within this two-sublattice description

$$w=\frac{1}{2i\pi}\oint dk\,\nabla_k\ln\left(-\frac{h_1(k)h_2(k)}{t_\perp}\right)=w_1+w_2\,. \tag{22}$$

Therefore, the wave functions are mainly polarised on sublattices A_1 and B_2 in the limit of low frequency $f \ll t_\perp$, and this two-sublattice picture captures entirely the band-structure topology of the Bloch Hamiltonian in Supplementary (12). As we show below, this has strong consequences for the elastic scattering problem in the two lowest frequency bands $\pm f_1(k)$.

4.3. Backscattering on the leftmost edge

The non-interacting Green function in the sublattice basis (A_1, A_2, B_1, B_2) is a 4×4 matrix satisfying

$$G(m,n)=\int_{-\pi}^{\pi}\frac{dk}{2\pi}\,[\omega\,\mathrm{I}_4-H(k)]^{-1}\,e^{ik(m-n)} \tag{23}$$

where the integers m and n label the unit cells, and I_4 is the 4×4 identity matrix. We model the leftmost edge by two infinite onsite potentials $V_1 = V_2$ in unit cell $m = 0$, and we focus on the standing-wave interference on unit cells $m \geq 1$ (see. Supplementary Fig. 4). Summing up the scattering diagrams in all orders in the onsite potentials leads to a T-matrix that generically reads

$$T=\begin{pmatrix}0&0&0&0\\0&0&0&0\\0&0&T_{B_1B_1}&T_{B_1B_2}\\0&0&T_{B_2B_1}&T_{B_2B_2}\end{pmatrix}\delta_{m,0}\,. \tag{24}$$

We aim to probe the winding number w and the bulk-boundary correspondence through the LDOS fluctuations on sublattice A_1. The standing-wave interference on sublattice A_1 are obtained from the scattering Green function

$$\Delta G_{A_1A_1}(m,m) = G_{A_1B_1}(m,0)T_{B_1B_1}G_{B_1A_1}(0,m) + G_{A_1B_1}(m,0)T_{B_1B_2}G_{B_2A_1}(0,m) \tag{25}$$
$$+ G_{A_1B_2}(m,0)T_{B_2B_1}G_{B_1A_1}(0,m) + G_{A_1B_2}(m,0)T_{B_2B_2}G_{B_2A_1}(0,m)\ . \tag{26}$$

To probe a larger winding number in the experiment, it is sufficient to focus on the situation where $t_1 = t_3$ and $t_2 = t_4$. This allows us to realise two different topological regimes in Supplementary Eq. (15), that are, $w = 0$ and $w = -2$. The transition is then driven by the single parameter t_1/t_2. The bare Green matrix of such a system satisfies

$$G(k,\omega) = [\omega\,\mathrm{I}_4 - H(k)]^{-1} \tag{27}$$
$$= \frac{1}{\omega^4 - \omega^2\left(2|h(k)|^2+\beta^2\right)+|h(k)|^4}\begin{pmatrix} \omega\left(\omega^2-|h(k)|^2-\beta^2\right) & \omega\beta h(k) & \left(\omega^2-|h(k)|^2\right)h(k) & \beta h^2(k) \\ \omega\beta h^*(k) & \omega\left(\omega^2-|h(k)|^2\right) & \omega^2\beta & \left(\omega^2-|h(k)|^2\right)h(k) \\ \left(\omega^2-|h(k)|^2\right)h^*(k) & \omega^2\beta & \omega\left(\omega^2-|h(k)|^2\right) & \omega\beta h(k) \\ \beta h^{*2}(k) & \left(\omega^2-|h(k)|^2\right)h^*(k) & \omega\beta h^*(k) & \omega\left(\omega^2-|h(k)|^2-\beta^2\right) \end{pmatrix}$$

where $h(k) = \alpha + e^{-ik}$ and we introduce $\alpha = t_1/t_2$ and $\beta = t_\perp/t_2$. So the frequency is in units of t_2. The space behaviour of the LDOS fluctuations on sublattice A_1 requires the knowledge of the real-space components $G_{A_1B_1}$ and $G_{A_1B_2}$ of the non-interacting Green matrix. These are obtained as

$$G_{A_1B_1}(m,0) = \int_{-\pi}^{\pi}\frac{dk}{2\pi}\frac{(\omega^2-|h(k)|^2)h(k)}{D(k)}e^{ikm} \tag{28}$$
$$G_{A_1B_2}(m,0) = \int_{-\pi}^{\pi}\frac{dk}{2\pi}\frac{\beta h^2(k)}{D(k)}e^{ikm}\ . \tag{29}$$

The poles provide the frequency bands $\pm f_1(k)$ and $\pm f_2(k)$ since

$$D(k) = \omega^4 - \omega^2\left(2|h(k)|^2+\beta^2\right)+|h(k)|^4 = \left(\omega^2-f_1^2(k)\right)\left(\omega^2-f_2^2(k)\right)\ . \tag{30}$$

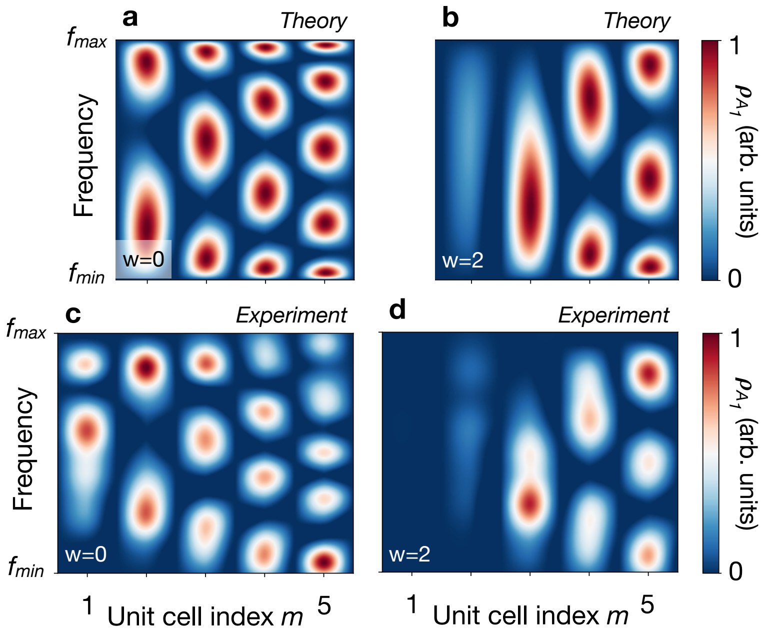

Supplementary Fig. 6. **Theoretical and experimental LDOS maps**: **a**, Theoretical space behaviour of the LDOS of the frequency band $-f_1$ on sublattice A_1 in the trivial regime $w = 0$ for $t_1/t_2 = 4.0$. **b**, Same as panel a but in the topological regime $w = -2$ for $t_1/t_2 = 0.25$. **c**, Experimental LDOS of the frequency band $-f_1$ measured on sublattice A_1 in the trivial regime $w = 0$ for $t_1 = 0.8t_\perp$ and $t_2 = 0.2t_\perp$. **d**, Same as panel c but in the topological regime $w = -2$ with $t_1 = 0.2t_\perp$ and $t_2 = 0.8t_\perp$. Note that the theoretical LDOS maps are obtained from Supplementary Eq. (34), which is valid if and only if $m \geq 2$. This is the reason why the site index $m = 1$ is not represented in panels a and b.

Furthermore, we have shown above that the energy bands $\pm f_1$ are always gapped from $\pm f_2$. Since only $\pm f_1$ can vanish at zero frequency, the different topological regimes can be fully characterised from the two frequency bands $\pm f_1$. In the limit $|h(k)| \ll \beta$, the frequency bands verify $f_1(k) \simeq |h(k)|$ and $f_2(k) \simeq \beta$. For unit cells satisfying $m \geq 2$, we find

$$G_{A_1B_1}(m,0) \simeq \int_{-\pi}^{\pi} \frac{dk}{2\pi} \frac{(\omega^2 - |h(k)|^2)h(k)}{(\omega^2 - |h(k)|^2)(\omega^2 - \beta^2)} e^{ikm} \simeq \oint_C \frac{dz}{2i\pi} \frac{\beta(\alpha z + 1)}{(\omega^2 - \beta^2)} z^{m-2} \simeq 0 \tag{31}$$

$$G_{A_1B_2}(m,0) \simeq \int_{-\pi}^{\pi} \frac{dk}{2\pi} \frac{\beta h^2(k)}{(\omega^2 - |h(k)|^2)(\omega^2 - \beta^2)} e^{ikm} \simeq \oint_C \frac{dz}{2i\pi} \frac{\beta(\alpha z + 1)^2}{(2\alpha z^2 + (1 + \alpha^2 - \omega^2)z + 2\alpha)(\beta^2 - \omega^2)^2} z^{m-2} \propto e^{ikm + i2\mathrm{Arg}[h(k)]} \,. \tag{32}$$

We have used the residue theorem for the complex variable $z = e^{ik}$ with respect to the unit circle C (see the appendix of Ref. [4] for more details). Importantly, the expressions above describe the elastic scattering for the frequency band $-f_1$ on unit cells $m \geq 2$. Otherwise, for the unit cell $m = 1$, there are extra poles inside C at $z = 0$. These must be taken into account to suitably describe the LDOS on site $m = 1$. They lead to additional terms in the right-hand side of Supplementary Eq. (32), so that $G_{A_1B_2}$ no longer relate only to $\mathrm{Arg}[h(k)]$. Such contributions vanish for unit cells $m \geq 2$, where we expect our real-space approach to directly reveal the topological winding of $\mathrm{Arg}[h(k)]$.

4.4. LDOS fluctuations and wavefront dislocation

The expressions above imply that the scattering processes described by $T_{B_2B_2}$ are dominant in Supplementary Eq. (25). This leads to

$$\Delta G_{A_1A_1}(m,m) \simeq G_{A_1B_2}(m,0) T_{B_2B_2} G_{B_2A_1}(0,m) \propto -ie^{i\varphi_{A_1}} \,, \tag{33}$$

where $\varphi_{A_1} = 2km + \delta_{A_1}(k) + \pi$ and the scattering phase shift is $\delta_{A_1}(k) = 4\mathrm{Arg}[h(k)]$. The space modulations of the LDOS in unit cells $m \geq 2$ then satisfy

$$\Delta\rho_{A_1}(m, \mathbf{s}) \propto \cos(2km + \delta_{A_1}(\mathbf{s}) + \pi) \,, \tag{34}$$

where we introduce the vector $\mathbf{s} = (t_1/t_2, k)$. This space behaviour of the LDOS is represented in Supplementary Fig. 6 and shows good agreements with the LDOS measured in the experiment on sites $m \geq 2$.

The number of interference fringes N_{A_1} on sublattice A_1 is also given by a sum rule over the frequency band $f_- = -f_1$

$$N_{A_1} = \frac{1}{2\pi} \int_{f_{\min}}^{f_{\max}} \frac{\partial \varphi_{A_1}}{\partial f_-} df_- = \int_0^{\pi} \frac{dk}{2\pi} \left(2m + \frac{\partial \delta_{A_1}}{\partial k} \right) = m + w \,. \tag{35}$$

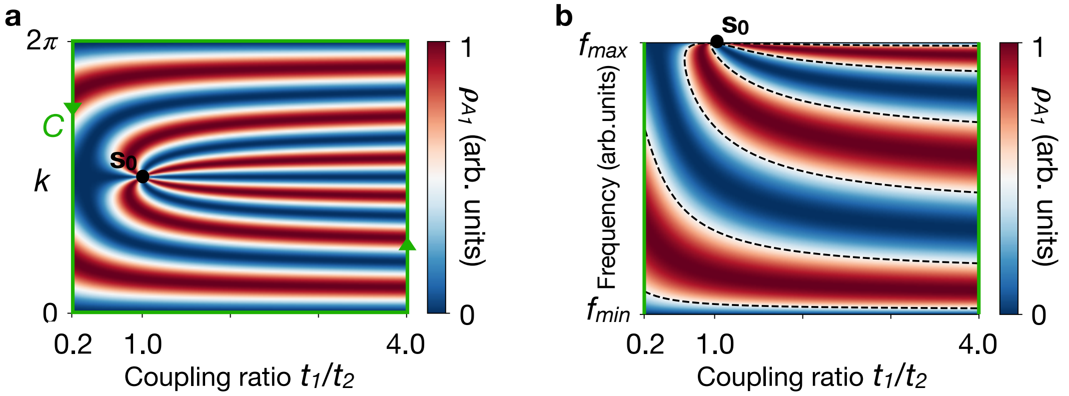

Supplementary Fig. 7. **Wavefront dislocation in the LDOS**: **a**, Theoretical LDOS fluctuations on sublattice A_1 as a function of vector $\mathbf{s} = (t_1/t_2, k)$ in 2D parameter space for site $m = 3$. The phase singularity in $\mathbf{s_0} = (1, \pi)$ at the topological phase transition is the source of a wavefront dislocation of strength $W_d = 4$ in the counter-clockwise Burgers circuit C. **b**, Theoretical LDOS fluctuations on sublattice A_1 in the 2D space $(t_1/t_2, -f_1(k))$, where $-f_1(0) = f_{\min}$ and $-f_1(0) = f_{\max}$. Two interference fringe emerges from the dislocation core in this frequency representation of the LDOS. This shows that the N_{A_1} shift in the interference fringes observed between $t_1/t_2 = 0.2$ and $t_1/t_2 = 4.0$ in Supplementary Fig. 6 is a direct measure of the dislocation strength.

The bulk-boundary correspondence further relates the variation of the scattering phase shift to the number of boundary modes as $\delta_{A_1}(0) - \delta_{A_1}(\pi) = \mathcal{N}_{A_1}$. Furthermore, Supplementary Fig. 7 shows that the homogeneous change observed in N_{A_1} is direct evidence of a wavefront dislocation of strength $W_{\mathrm{d}} = 4$ at the topological transition

$$2\pi W_{\mathrm{d}} = \oint_C \nabla_{\mathbf{s}} \varphi_{A_1}(\mathbf{s}) \cdot \mathbf{ds} = 8\pi \,, \tag{36}$$

whereas $W_{\mathrm{d}} = 2$ for the SSH chain. This demonstrates that wavefront dislocations can also reveal topological transitions involving winding numbers larger than 1.

---

[*] clement.dutreix@u-bordeaux.fr

[†] fabrice.mortessagne@univ-cotedazur.fr

[1] Bellec, M., Kuhl, U., Montambaux, G. & Mortessagne, F. Tight-binding couplings in microwave artificial graphene. *Physical Review B* **88**, 115437 (2013).

[2] Dutreix, C. *Impurity and boundary modes in the honeycomb lattice*. Ph.D. thesis, Paris 11 (2014).

[3] Xie, D., Gou, W., Xiao, T., Gadway, B. & Yan, B. Topological characterizations of an extended Su–Schrieffer–Heeger model. *npj Quantum Information* **5**, 1–5 (2019).

[4] Dutreix, C. & Delplace, P. Geometrical phase shift in Friedel oscillations. *Physical Review B* **96**, 195207 (2017).
